# Molecular regulation of ERK5 in development of diabetic retinopathy

**DOI:** 10.18632/oncotarget.23392

**Published:** 2017-12-17

**Authors:** Qi Zhao, Lin Wang, Yuan Sun, Xiao-Xia Wang

**Affiliations:** ^1^ Department of Ophthalmology, The Second Hospital of Dalian Medical University, Dalian 116023, China

**Keywords:** diabetic retinopathy (DR), ERK5, streptozocin (STZ), retina neovascularization

## Abstract

Diabetic retinopathy (DR) is a major complication of diabetes, and causes pathological changes in retina blood vessels, as the most common cause of vision loss. Extracellular-signal-regulated kinase 5 (ERK5) is the newest discovered member in the mitogen-activated protein kinases (MAPKs) family, and recent evidence demonstrates an essential role of ERK5 signaling in the angiogenesis. However, whether ERK5 signaling may regulate DR development is unknown. Here, we used a streptozocin (STZ)-induce mouse DR model to investigate this question. We detected significant increases in the phosphorylation of ERK5, a signature of ERK5 activation in the purified retinal endothelial cells in DR mice, compared to control mice. *In vivo* suppression of ERK5 phosphorylation through administration of a specific inhibitor of ERK5 activation, BIX02189, did not prevent the occurrence of STZ-induced diabetes in mice, but significantly alleviated the severity of DR, seemingly through attenuating the retina neovascularization. Thus, our study suggests a previously unappreciated role of ERK5 signaling in DR development.

## INTRODUCTION

Major complications of diabetes, a prevalent chronic disease characterized with impaired glucose metabolism, can be either macroangiopathy or microangiopathy [1]. Macroangiopathy is an advanced stage of atherosclerosis, while microangiopathy includes diabetic retinopathy (DR) and nephropathy [2]. The major pathological changes in DR are appearance of abnormal retina blood vessels, which appear to be dysregulated, condensed and fragile [2]. Since severe DR may lead to vision loss and blindness in the diabetes patients, prevention and treatment of early abnormal retina vascularization are thus the most important and effective strategies in DR therapy [3].

Extracellular-signal-regulated kinase 5 (ERK5) is a new member in the mitogen-activated protein kinases (MAPKs) family, and was discovered after the extracellular signal-regulated kinases (ERK1/2), the p38 kinases, and the Jun amino-terminal kinases (JNKs) [4]. Among all MAPK members, ERK5 is the largest MAPK and is ubiquitously expressed [4]. ERK5 is exclusively activated by its upstream kinase MEK5 at presence of some growth factors like granulocyte colony-stimulating factor (G-CSF), nerve growth factor (NGF), fibroblast growth factor (FGF), and platelet-derived growth factor (PDGF), or in the circumstance of oxidative and hyperosmotic stress [4]. MEK5 phosphorylates ERK5 at the its C-terminal, leading to the dissociation of the phosphorylated ERK5 (pERK5) from the complex that it combines with Hsp90 and Cdc37, and the nuclear translocation of pERK5 [4]. Hence, suppression of MEK5 specifically inhibits activation of ERK5. Recently, it was found that constitutive MEK5 suppressed SUMOylation of ERK5 to protect heart function in diabetic mice [5]. Moreover, adipocyte-specific ERK5-deficient mice exhibited elevated blood glucose and compromised insulin sensitivity [6].

Hence, we aimed to investigate whether ERK5 signaling may control development of DR through its regulation of pathological angiogenesis. We used a streptozocin (STZ)-induce mouse DR model, in which we detected significant increases in the pERK5 in the purified retinal endothelial cells in DR mice, compared to control mice. *In vivo* suppression of ERK5 phosphorylation through administration of a specific inhibitor of ERK5 activation, BIX02189, did not prevent the occurrence of STZ-induced diabetes in mice, but significantly alleviated the severity of DR, seemingly through attenuating the retina neovascularization.

## RESULTS

### Induction of diabetes in mice by STZ administration

**Figure 1:**
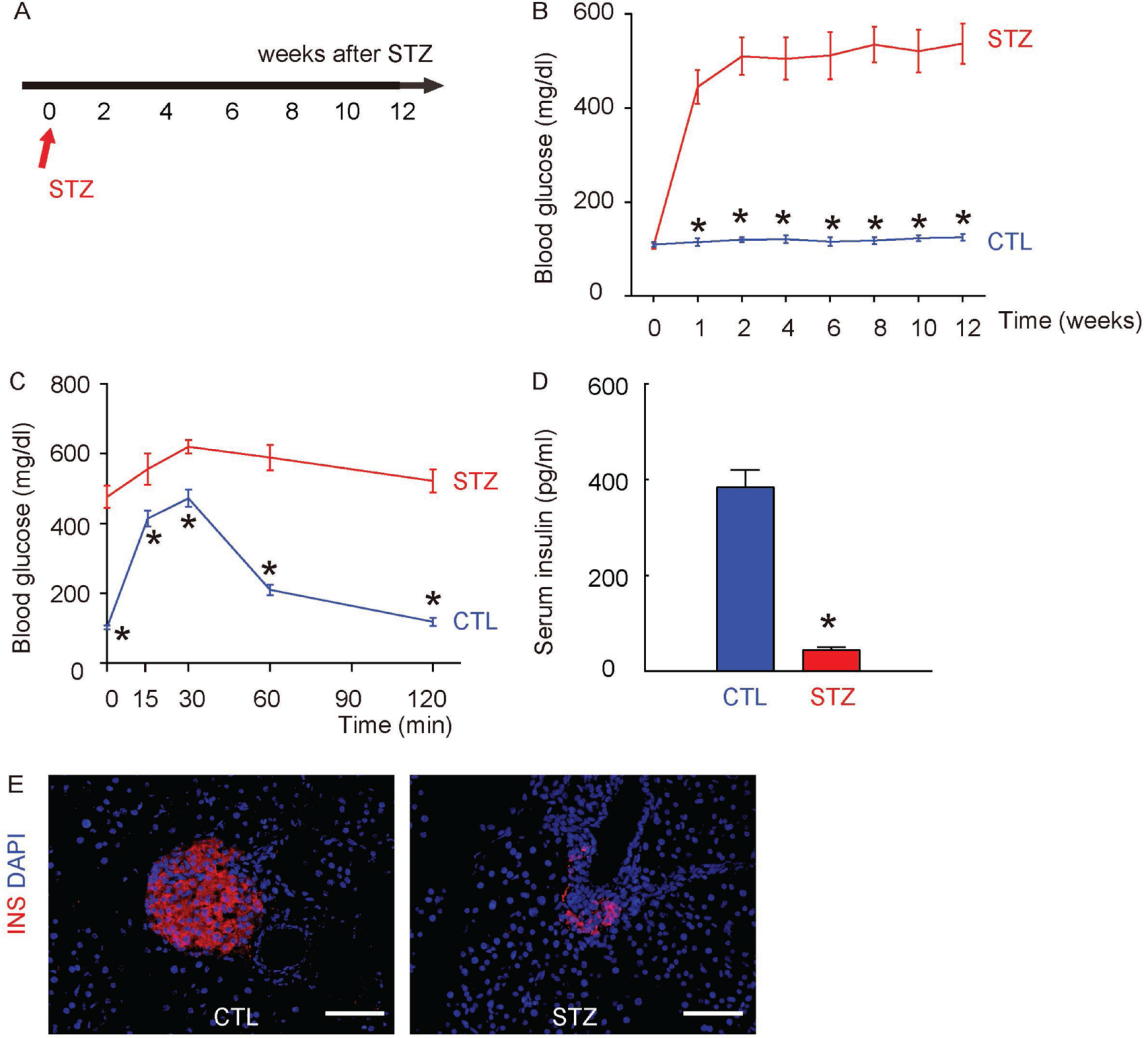
Induction of diabetes in mice by STZ administration (**A**) A single intraperitoneal injection of beta cell toxin STZ was used to induce diabetes (STZ) and DR in mice. The control mice (CTL) received an intraperitoneal injection of an equivalent volume of normal saline at the same time of STZ administration. The mice were followed up for 12 weeks. (**B**) Fasting blood sugar. (**C**) IPGTT. (**D**) Serum insulin content. (**E**) Representative immunostaining for insulin (INS) on pancreas sections at 12 weeks after STZ. DAPI: nuclear staining. ^*^p < 0.05. *N =* 10. Scale bars are 50 µm.

We used a single intraperitoneal injection of beta cell toxin STZ to induce diabetes and DR in mice. The control mice received an intraperitoneal injection of an equivalent volume of normal saline at the same time of STZ administration. The mice were followed up for 12 weeks (Figure 1A). We found that after 1 week of STZ injection, the mice developed significantly higher fasting blood sugar (Figure 1B), significantly impaired glucose tolerance at 12 weeks after STZ (Figure 1C) and significantly lower serum insulin content at 12 weeks after STZ (Figure 1D), compared to the control mice. The insulin staining on mouse pancreas sections at 12 weeks after STZ showed loss of the majority of islet beta cells in STZ mice, compared to the control mice (Figure 1E). Thus, STZ induces diabetes in mice.

### Induction of DR in mice by STZ administration

**Figure 2:**
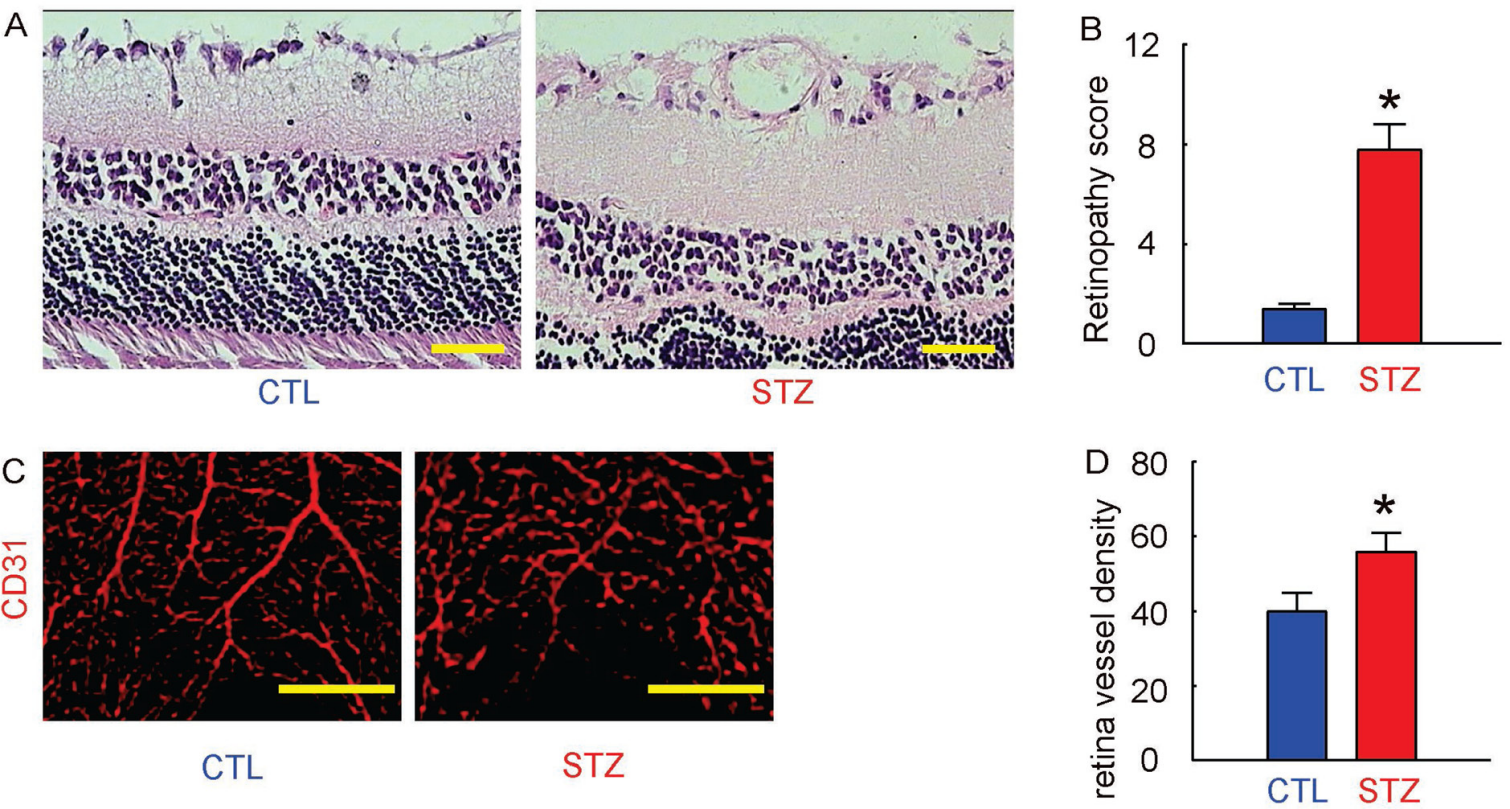
Induction of DR in mice by STZ administration (**A**) Representative histological images of mouse retina at 12 weeks after STZ. (**B**) Retinopathy score. (**C**) Representative CD31 staining of mouse retina at 12 weeks after STZ. (**D**) Retina vessel density. ^*^p < 0.05. *N =* 10. Scale bars are 50 µm.

Next, we examined whether STZ treatment had induced DR in these mice. We found that 12 weeks after STZ, the retinopathy score in STZ mice was significantly increased, compared to the control mice, shown by representative histological images (Figure 2A), and by quantification (Figure 2B). The CD31 staining was performed on retinal tissue, shown by representative images (Figure 2C). We found that the vessel density in eyes in STZ mice was also significantly increased, compared to the control mice (Figure 2D). Together, these data suggest that STZ treatment induced DR in these mice in 12 weeks.

### ERK5 signaling is activated in retinal endothelial cells in STZ mice

**Figure 3:**
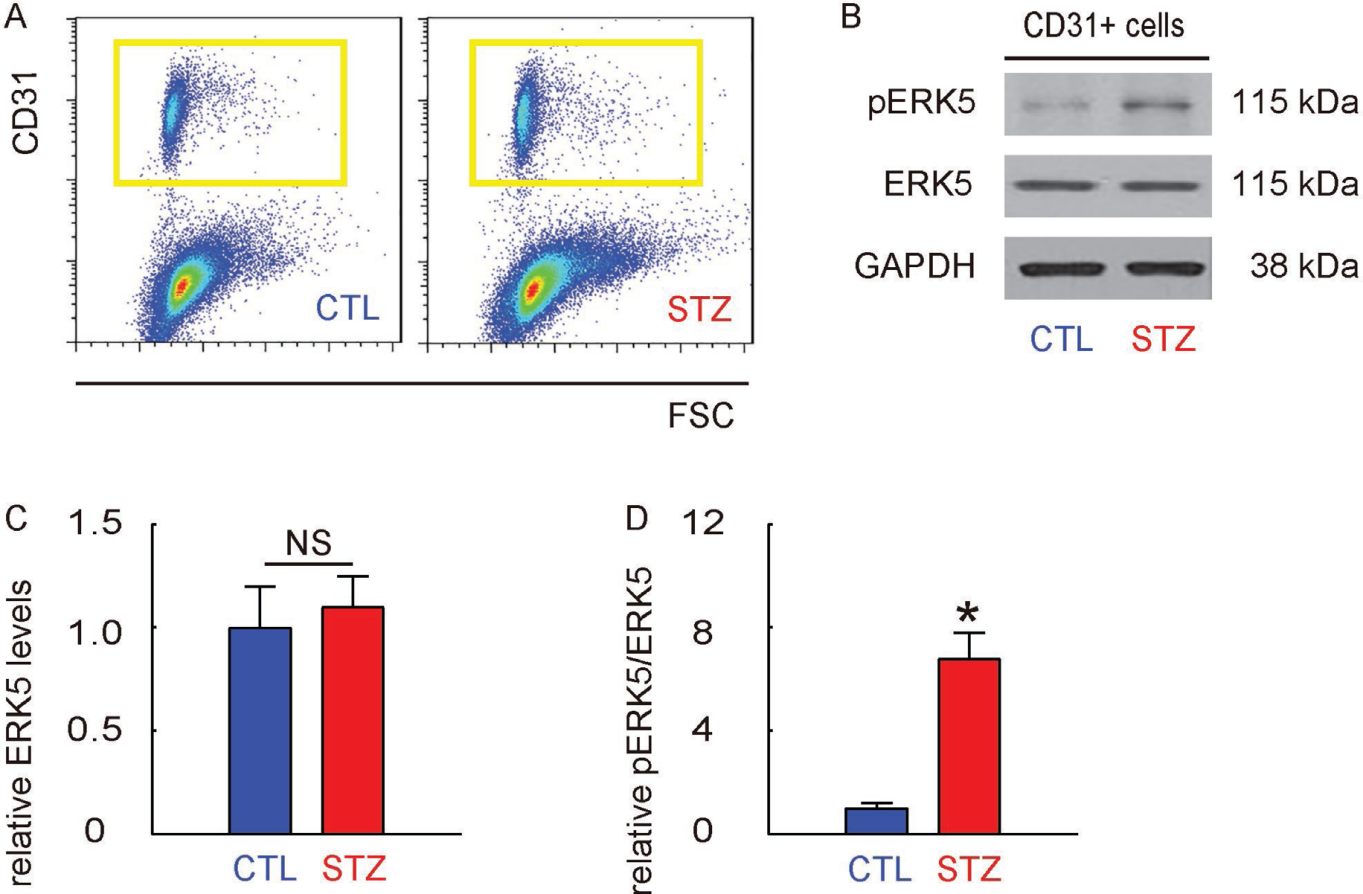
ERK5 signaling is activated in retinal endothelial cells in STZ mice (**A**) Mouse retina were thus isolated and dissociated into single cells for flow cytometry-based purification of CD31+ retinal endothelial cells, shown by representative flow charts. (**B**) Representative Western blotting images of ERK5 and phosphorylated ERK5 (pERK5) in CD31+ retinal endothelial cells. (**C**) Quantification of ERK5 levels (normalized to GAPDH). (**D**) Quantification of pERK5 levels (normalized to ERK5). ^*^p < 0.05. NS: non-significant. *N =* 10.

Mouse retina were thus isolated and dissociated into single cells for flow cytometry-based purification of CD31+ retinal endothelial cells, shown by representative flow charts (Figure 3A). The phosphorylation of ERK5 in CD31+ retinal endothelial cells was determined by Western blotting, shown by representative images (Figure 3B). We found that the total ERK5 in retinal endothelial cells was unaltered (Figure 3C), while the phosphorylated ERK5 (pERK5) was significantly increased in retinal endothelial cells from STZ mice, compared to the control mice (Figure 3D). Thus, ERK5 signaling is activated in retinal endothelial cells in STZ mice.

### Suppression of ERK5 activation does not alter diabetes induction by STZ in mice

**Figure 4:**
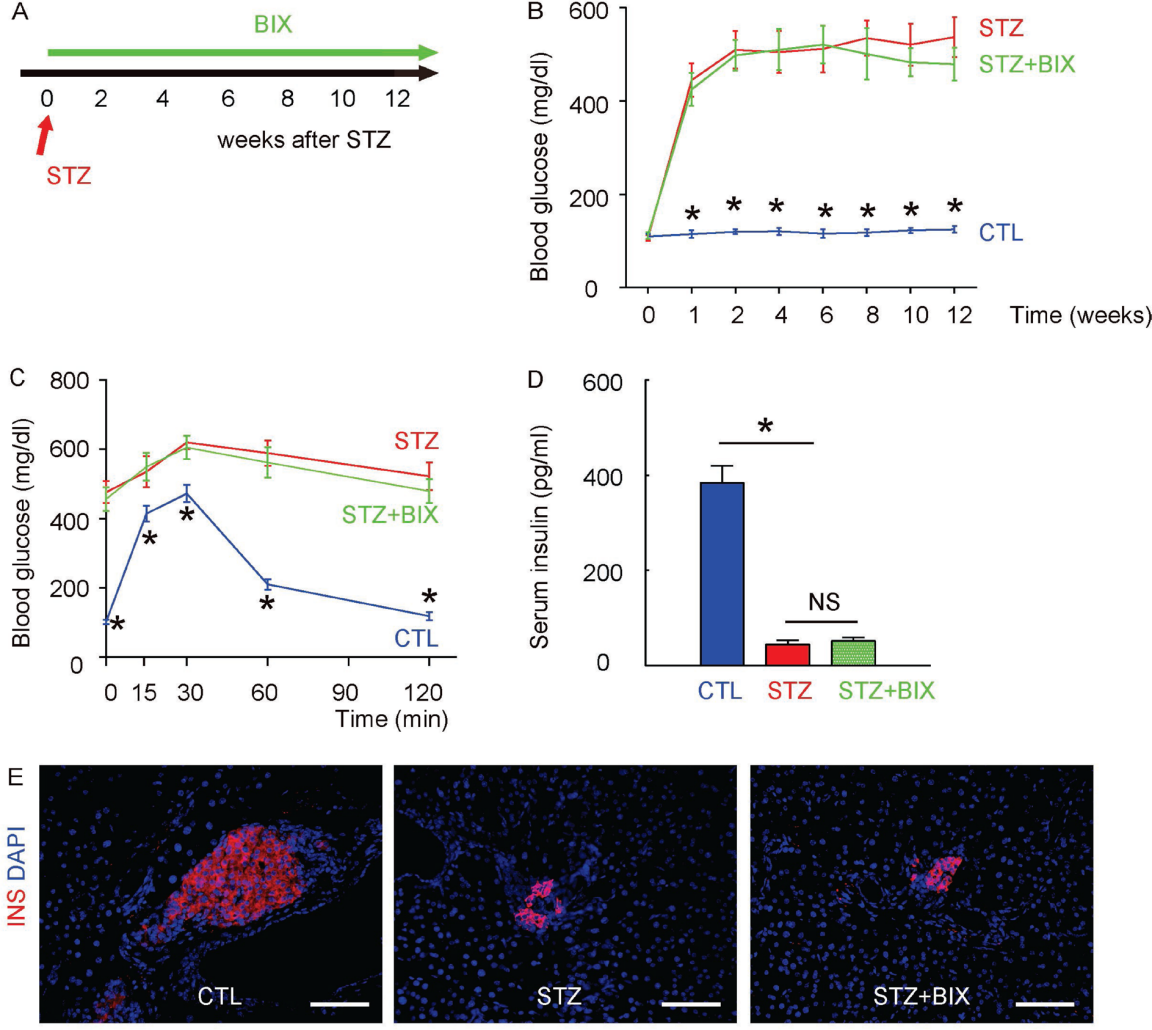
Suppression of ERK5 activation does not alter diabetes induction by STZ in mice (**A**) A single intraperitoneal injection of beta cell toxin STZ was used to induce diabetes and DR in mice. Intraperitoneal injections of a specific inhibitor of ERK5 phosphorylation, BIX02189, in a twice-per-week frequency, into the STZ-treated mice for 12 weeks (STZ+BIX) were done. STZ-mice that received intraperitoneal injections of an equivalent volume of normal saline at the same frequency were used as controls (STZ). The control mice (CTL) received an intraperitoneal injection of an equivalent volume of normal saline at the same time of STZ administration and received no BIX injections. The mice were followed up for 12 weeks. (**B**) Fasting blood sugar. (**C**) IPGTT. (**D**) Serum insulin content. (**E**) Representative immunostaining for insulin (INS) on pancreas sections at 12 weeks after STZ. DAPI: nuclear staining. ^*^p < 0.05. NS: non-significant. *N =* 10. Scale bars are 50 µm.

In order to evaluate the role of ERK5 activation in the development of DR, we did intraperitoneal injections of a specific inhibitor of ERK5 phosphorylation, BIX02189, in a twice-per-week frequency, into the STZ-treated mice for 12 weeks (STZ+BIX). STZ-mice that received intraperitoneal injections of an equivalent volume of normal saline at the same frequency were used as controls (STZ). The mice were followed up for 12 weeks (Figure 4A). We found that the development of high fasting blood sugar (Figure 4B), impaired glucose tolerance at 12 weeks after STZ (Figure 4C) and low serum insulin content at 12 weeks after STZ (Figure 4D) in STZ+BIX mice was not different from STZ mice. The insulin staining on mouse pancreas sections 12 weeks (the time of sacrifice of the mice) after STZ also showed that the loss of the majority of islet beta cells by STZ was not altered by BIX administration (Figure 4E). Thus, suppression of ERK5 activation does not alter diabetes induction by STZ in mice.

### Activation of ERK5 signaling in retinal endothelial cells by STZ is suppressed by BIX

**Figure 5:**
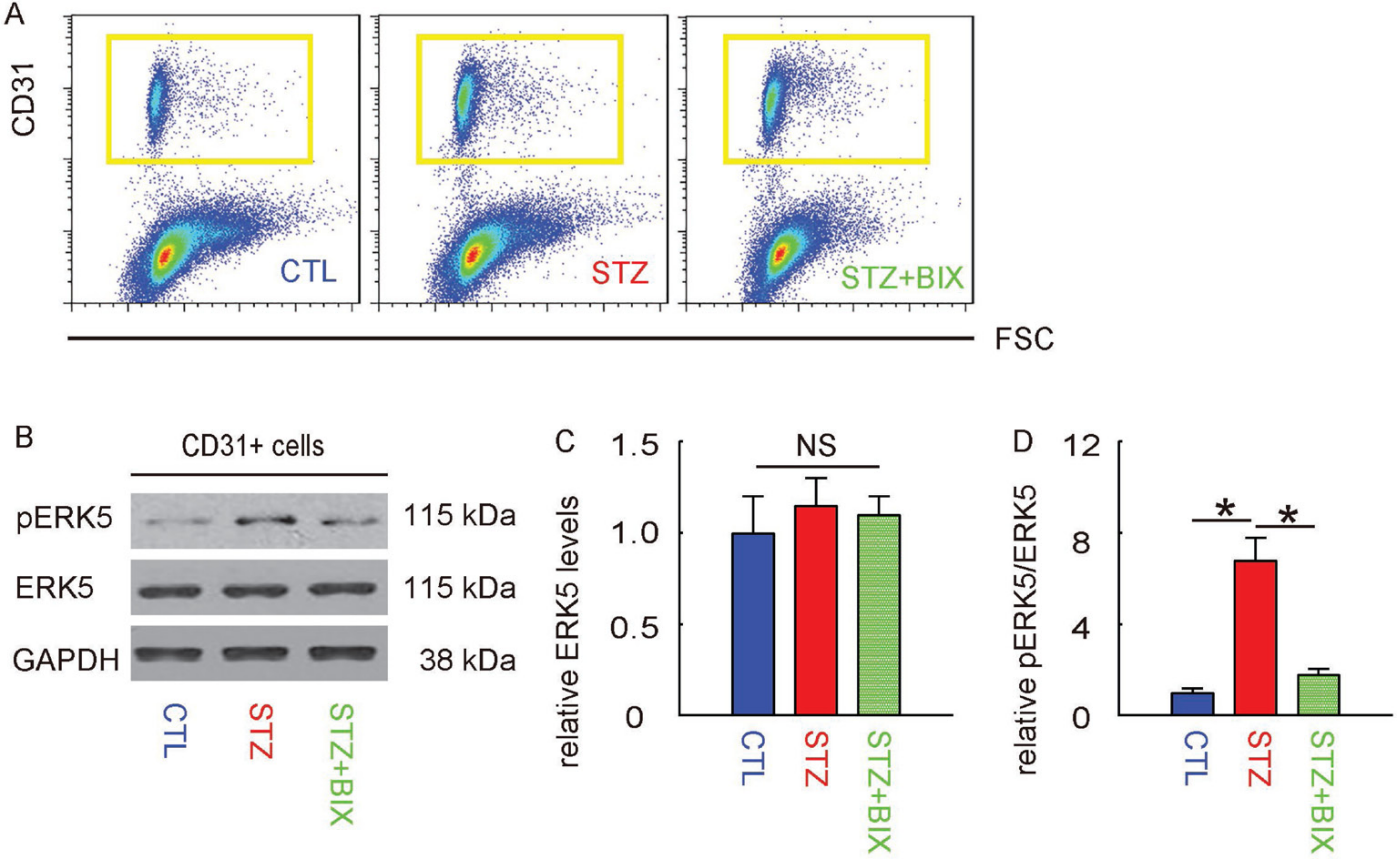
Activation of ERK5 signaling in retinal endothelial cells by STZ is suppressed by BIX (**A**) Mouse retina were thus isolated and dissociated into single cells for flow cytometry-based purification of CD31+ retinal endothelial cells, shown by representative flow charts. (**B**) Representative Western blotting images of ERK5 and phosphorylated ERK5 (pERK5) in CD31+ retinal endothelial cells. (**C**) Quantification of ERK5 levels (normalized to GAPDH). (**D**) Quantification of pERK5 levels (normalized to ERK5). ^*^p < 0.05. NS: non-significant. *N =* 10.

At 12 weeks after treatments, mouse retina were thus isolated and dissociated into single cells for flow cytometry-based purification of CD31+ retinal endothelial cells, shown by representative flow charts (Figure 5A). The phosphorylation of ERK5 in CD31+ retinal endothelial cells was determined by Western blotting, shown by representative images (Figure 5B). We found that the total ERK5 in retinal endothelial cells was unaltered in all 3 groups (Figure 5C), while the upregulation of pERK5 levels in retinal endothelial cells by STZ was significantly attenuated by BIX (Figure 5D). Thus, activation of ERK5 signaling in retinal endothelial cells by STZ is suppressed by BIX,

### BIX attenuates DR in STZ-mice

**Figure 6:**
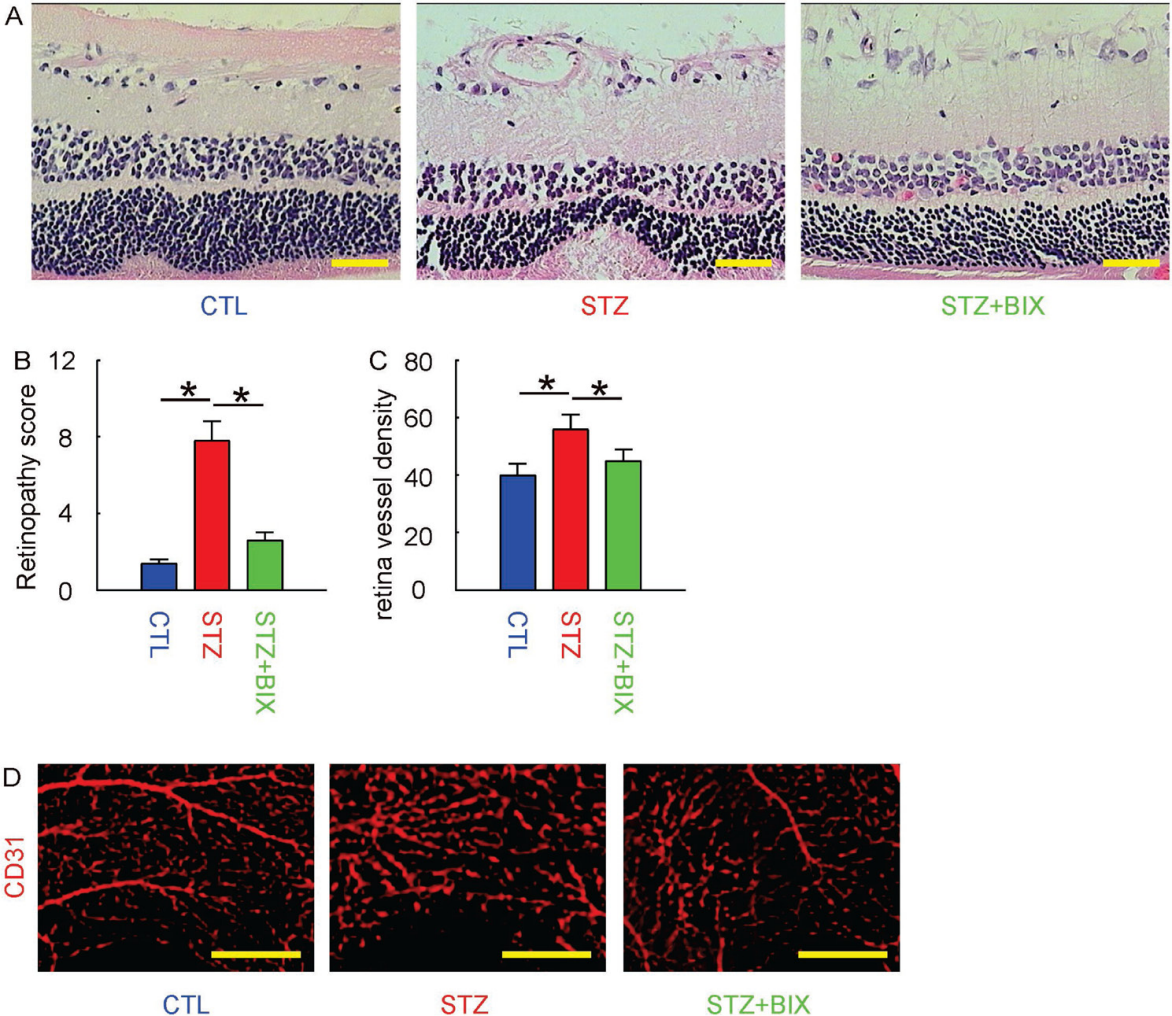
BIX attenuates DR in STZ-mice (**A**) Representative histological images of mouse retina at 12 weeks after STZ. (**B**) Retinopathy score. (**C**) Retina vessel density. (**D**) Representative CD31 staining of mouse retina at 12 weeks after STZ. ^*^p < 0.05. *N =* 10. Scale bars are 50 µm.

Finally, we examined whether BIX may affect the severity of DR in STZ-treated mice. We found that 12 weeks after treatments, the increases in retinopathy score by STZ were significantly attenuated by BIX, shown by representative histological images (Figure 6A), and by quantification (Figure 6B). The CD31 staining was performed on retinal tissue, and we found that the increases in vessel density in eyes by STZ were also significantly attenuated (Figure 6C). The representative images for CD31 staining were shown (Figure 6D). Together, these data suggest that suppression of ERK5 signaling attenuates DR in STZ-mice.

## DISCUSSION

ERK5 is known to be highly expressed in the endothelial cells and plays an essential role in the maintenance of endothelial function and blood vessel integrity [7]. ERK5 knockout mice die at embryonic day 10 due to cardiovascular defects [8]. Moreover, it appears that ERK5 is critical for endothelial cell function and that ERK5-depleted mice exhibit abnormal heart development as a consequence of endothelial cell dysfunction [9]. Moreover, ERK5 is critical to keep vascular integrity, since mice with induced ERK5 depletion display multi-organ hemorrhages [9].

In the current study, in the purified retinal endothelial cells in DR mice, we detected significant increases in the phosphorylation of ERK5, which is a signature of ERK5 activation. Indeed, previous studies have shown that ERK5 is activated by dual phosphorylation at Thr218/Tyr220 by its upstream kinase regulator, MEK5, and MEK5 first phosphorylates ERK5 on Thr218, which induce the subsequent phosphorylation of Tyr220 [10]. In addition, active ERK5 undergoes auto-phosphorylation on the C-terminal at Thr28, Ser421, Ser433, Ser496, Ser731 and Thr733, which further enhances ERK5 transcriptional activity [10]. Thus, based on our data and these previous publications, we hypothesized that ERK5 signaling may play a role in development of DR, likely through retinal vascularization.

To prove it, we gave mice a specific inhibitor of ERK5 activation, BIX02189 [11], to specifically suppress ERK5 signaling. BIX02189 has been well characterized as a specific inhibitor for the catalytic function of MEK5, which is required for the phosphorylation of ERK5, but not phosphorylation of ERK1/2 in cells [11]. We found that use of BIX02189 did not prevent the occurrence of STZ-induced diabetes in mice, but significantly alleviated the severity of DR, seemingly through attenuation of the retina neovascularization. This result confirmed our hypothesis. The underlying mechanism underlying the effects of ERK5 signaling on retinal vascularization may stem from its protective effects against endothelial cell apoptosis [10, 12], which results from shear stress during the pathogenic initiation of DR [12]. On the other hand, the stimuli of ERK signaling may be a summary of different growth factors in DR, including G-CSF, NGF, FGF, PDGF, and vascular endothelial growth factor (VEGF) [4].

The role of ERK5 signaling in the pathogenesis of DR has been recently reported by Chakrabarti group. They have found that ERK5 inactivation results in augmented VEGF mRNA expression which contributes to DR-associated neovascularization in rats [13]. Moreover, they have shown that ERK5 signaling may play a role in the glucose-induced increases in fibronectin production in DR [14]. Together, these studies and our work highlight a potential role of ERK5 signaling in the pathogenesis of DR. Future studies may examine the ERK5 and its activated form in patients’ specimens to figure out whether ERK5 signaling is similarly important in DR development in humans, like in rodents.

## MATERIALS AND METHODS

### Mouse treatment

All mouse experiments were approved by the Animal Research and Care Committee at the First Hospital of the Second Hospital of Dalian Medical University. Female C57BL/6 mice (SLAC Laboratory Animal Co. Ltd, Shanghai, China) were used in the experiments at 10 weeks of age. The beta cell toxin STZ (Sigma-Aldrich, St Louis, MO, USA) was injected from the tail vein at 150 mg/kg body weight. The ERK5 inhibitor BIX02189 (Selleck Chemicals, Houston, TX, USA) [11] was intraperitoneally injected into the mice at a dose of 10 mg/kg body weight in 100 ml saline since the day of STZ injection, at a frequency of twice per week, through the end of experiment at 12 weeks after STZ. The control mice received the same volume of normal saline at the same frequency. Fasting blood glucose, intraperitoneal glucose tolerance test (IPGTT) and serum insulin content were measured as described [15]. Briefly, the mice were fasted overnight before measurement of blood sugar was performed at 9am via tail tip puncture using a glucometer (Accu-Chek, Roche, Nutley, NJ, USA). To examine the possible glucose tolerance, after a 16 hour fasting, the mice received an intraperitoneal injection of 2 mg/g body weight of dextrose, after which blood samples were obtained by tail clipping at 0, 15, 30, 60 and 120 min. Serum insulin was measured with an insulin ELISA kit (R&D System, Los Angeles, CA, USA).

### Retinal epithelial cell isolation

Mouse retina were dissected out and dissociated into single cells by incubation with 0.35 mg/ml collagenase IV (Sigma-Aldrich, St. Louis, MO, USA) for 30 minutes. The single cell retinal cell population was then incubated with FTIC-conjugated rat anti-mouse CD31 antibody (Becton-Dickinson Biosciences, San Jose, CA, USA) for 20 minutes before being analyzed and sorted by flow cytometry.

### Retinopathy grading

Mouse retina sections received H&E staining. Retinopathy grade was assessed by two experienced investigators using the scoring system as described [16]. This scoring system assesses a summary of blood vessel growth, blood vessel tufts, central vasoconstriction, extra-retinal neovascularization, retinal hemorrhage and degree of vessel tortuosity.

### Immunohistochemistry for insulin and CD31

Tissues were fixed in 4% paraformaldehyde at 4^°^C for 4 hours, followed by cryoprotection in 30% sucrose overnight before snap-freezing. Slides of 6µm-thickness was performed during sectioning of the samples. Primary antibodies used in this study were: Guinea pig anti-insulin (DAKO, Carpinteria, CA, USA) and rat polyclonal anti-CD31 (Becton-Dickinson Biosciences). The secondary antibodies were Cy3-conjugated anti-guinea pig or anti-rat secondary antibodies generated from donkey (Jackson ImmunoResearch Labs, West Grove, PA, USA). Nucleus staining was performed with 4,6-Diamidino-2-phenylindole (DAPI, Becton-Dickinson Biosciences, San Jose, CA, USA).

### Retina angiography and vessel density

Mouse retinal vessels were stained for CD31 as abovementioned. Vessel density was determined as CD31+ area divided by total tissue area using Image J (NIH, Bethesda, MA, USA).

### Western blotting

Protein was extracted using RIPA buffer (Sigma-Aldrich) for Western Blot. Protein concentration was measured with using BCA assay (DAKO). Primary antibodies for Western blot are rabbit anti-ERK5 (Cell signaling, San Jose, CA, USA), rabbit anti-pERK5 (Cell signaling) and rabbit anti-GAPDH (Santa Cruz Biotechnology, Dallas, Texas, USA). Secondary antibody is HRP-conjugated anti-rabbit antibody (Jackson ImmunoResearch Labs). Images shown in the figure were representative from 5 repeats. Densitometry of Western blots was quantified with Image J software (NIH, Bethesda, MA, USA).

### Statistical analysis

All statistical analyses were carried out using GraphPad prism 6.0 (GraphPad Software Inc. La Jolla, CA, USA). All values are depicted as mean ± SD and are considered significant if *p* < 0.05. All data were statistically analyzed using one-way ANOVA with a Bonferroni correction, followed by Fisher’s Exact Test for comparison of two groups.
